# First-site-metastasis pattern in patients with inoperable stage III NSCLC treated with concurrent chemoradiotherapy with or without immune check-point inhibition: a retrospective analysis

**DOI:** 10.1007/s00066-023-02175-6

**Published:** 2023-11-17

**Authors:** Kerstin Hofstetter, Julian Taugner, Lukas Käsmann, Sina Mansoorian, Benedikt Flörsch, Chukwuka Eze, Amanda Tufman, Niels Reinmuth, Thomas Duell, Claus Belka, Farkhad Manapov

**Affiliations:** 1 Department of Radiation Oncology, University Hospital, LMU Munich, Munich, Germany; 2 Comprehensive Pneumology Center Munich (CPC-M), Member of the German Center for Lung Research (DZL), Munich, Germany; 3 German Cancer Consortium (DKTK), Partner Site Munich, Munich, Germany; 4 Division of Respiratory Medicine and Thoracic Oncology, Department of Internal Medicine V, Thoracic Oncology Centre Munich, LMU Munich, Munich, Germany; 5 Asklepios Kliniken GmbH, Asklepios Fachkliniken Muenchen, Gauting, Germany

**Keywords:** Lung Cancer, Thoracic Radiotherapy, Outcome, Immune Checkpoint Inhibition, Metastasis

## Abstract

**Purpose:**

The aim of this study was to investigate a first-site-metastasis pattern (FSMP) in unresectable stage III NSCLC after concurrent chemoradiotherapy (cCRT) with or without immune checkpoint inhibition (ICI).

**Methods:**

We defined three patient subgroups according to the year of initial multimodal treatment: A (2011–2014), B (2015–2017) and C (2018–2020). Different treatment-related parameters were analyzed. Observed outcome parameters were brain metastasis-free survival (BMFS), extracranial distant metastasis-free survival (ecDMFS) and distant metastasis-free survival (DMFS).

**Results:**

136 patients treated between 2011 and 2020 were included with ≥ 60.0 Gy total dose and concurrent chemotherapy (cCRT); thirty-six (26%) received ICI. Median follow-up was 49.7 (range:0.7–126.1), median OS 31.2 (95% CI:16.4–30.3) months (23.4 for non-ICI vs not reached for ICI patients, *p* = 0.001).

Median BMFS/ecDMFS/DMFS in subgroups A, B and C was 14.9/16.3/14.7 months, 20.6/12.9/12.7 months and not reached (NR)/NR/36.4 months (*p* = 0.004/0.001/0.016).

For cCRT+ICI median BMFS was 53.1 vs. 19.1 months for cCRT alone (*p* = 0.005). Median ecDMFS achieved 55.2 vs. 17.9 (*p* = 0.003) and median DMFS 29.5 (95% CI: 1.4–57.6) vs 14.93 (95% CI:10.8–19.0) months (*p* = 0.031), respectively.

Multivariate analysis showed that age over 65 (HR:1.629; *p* = 0.036), GTV ≥ 78 cc (HR: 2.100; *p* = 0.002) and V20 ≥ 30 (HR: 2.400; *p* = 0.002) were negative prognosticators for BMFS and GTV ≥ 78 cc for ecDMFS (HR: 1.739; *p* = 0.027).

After onset of brain metastasis (BM), patients survived 13.3 (95% CI: 6.4–20.2) months and 8.6 months (95% CI: 1.6–15.5) after extracranial-distant-metastasis (ecDM). Patients with ecDM as FSMP reached significantly worse overall survival of 22.1 (range:14.4–29.8) vs. 40.1 (range:18.7–61.3) months (*p* = 0.034) in the rest of cohort. In contrast, BM as FSMP had no impact on OS.

**Conclusion:**

This retrospective analysis of inoperable stage III NSCLC patients revealed that age over 65, V20 ≥ 30 and GTV ≥ 78 cc were prognosticators for BMFS and GTV ≥ 78 cc for ecDMFS. ICI treatment led to a significant improvement of BMFS, ecDMFS and DMFS. ecDM as FSMP was associated with significant deterioration of OS, whereas BM as FSMP was not.

**Supplementary Information:**

The online version of this article (10.1007/s00066-023-02175-6) contains supplementary material, which is available to authorized users.

## Introduction

Regarding cancer-related mortality, lung cancer is the most common cause worldwide [1]. Inoperable stage III NSCLC is a highly heterogeneous disease with varying macroscopic tumor extensions, and therefore, patients’ prognosis depends heavily on multiple patient and treatment factors. After multimodality treatment, historically, the five-year survival rate of patients has been as low as ten to thirty percent [2–4]. The standard of care for patients with unresectable stage III non-small cell lung cancer (NSCLC) is concurrent chemoradiotherapy [5] followed by maintenance therapy with the programmed cell death ligand 1 (PD-L1) inhibitor durvalumab [6]. The addition of immune checkpoint inhibition (ICI) to the multimodality treatment strategy has led to a significant improvement in patient outcomes, as demonstated by the unprecedented results of the PACIFIC trial [7, 8].

However, distant failure remains a burden for patients with unresectable NSCLC: Over time 30% of affected patients develop brain metastases (BM) [9], but there is encouraging evidence that anti-PD-1/PD-L1 agents may have a beneficial impact on their development [10]. Knowledge of the effects of chemoradioimmunotherapy on the development of distant metastases (DM) is limited. This retrospective analysis evaluates the impact of treatment patterns and patient-related factors on brain metastasis-free (BMFS), extracranial distant metastasis-free (ecDMFS), and distant metastasis-free survival (DMFS) after concurrent chemoradiotherapy (cCRT) with and without immune checkpoint inhibitors (ICI).

## Patients and methods

A total of 189 consecutive patients with stage III NSCLC were assessed for eligibility in 2021. The retrospective study and analysis of individual patient data were approved by the local ethics committee (reference number: 17-230). Informed consent to the treatment and data collection for research purposes was obtained from all patients.

Out of the 189 patients treated between February 2011 and November 2020, 136 (71.9%) were included. All selected patients had been diagnosed with unresectable stage III A–C (UICC 8th edition) NSCLC. They received concurrent cCRT with and without ICI. This included either simultaneous and maintenance treatment with the PD‑1 inhibitor nivolumab as part of the phase II ETOP 6–14 NICOLAS study [11, 12], or maintenance treatment with the PD-L1 inhibitor durvalumab in accordance with the PACIFIC trial [7].

The cCRT consisted of conventional fractionated thoracic radiotherapy (TRT) with a total dose of ≥ 60 Gy and platinum-based chemotherapy (cisplatin/carboplatin in combination with vinorelbine/pemetrexed). To assess improvements over time, three subgroups of patients were classified according to the year of the first TRT: Subgroup A (2011–2014), Subgroup B (2015–2017), and Subgroup C (2018–2020).

All survival parameters were calculated from the last day of TRT only considering the first site of failure (FSMP), including brain metastasis-free survival (BMFS), extracranial distant metastasis-free survival (ecDMFS) and distant metastasis-free survival (DMFS). Patients with local recurrence prior to distant failure or with simultaneous multifocal progression were not included in the BM, ecDM or DM subgroup, Patient and treatment-related characteristics are displayed in Table 1.

**Table 1:** Patient characteristics

Entire cohort		DM	BM	ecDM
N (%)	136 (100)	61 (45)	21 (15)	41 (30)
*Year of TRT*				
Subgroup A: 2011–2014	37 (27)	16 (43)	8 (22)	9 (24)
Subgroup B: 2015–2017	49 (36)	25 (51)	3 (6)	22 (45)
Subgroup C: 2018–2020	50 (37)	20 (40)	10 (20)	10 (20)
*Gender*				
Female	43 (32)	22 (51)	12 (28)	11 (26)
Male	93 (68)	39 (42)	9 (10)	30 (32)
*T category*				
1–2	33 (24)	17 (51)	5 (15)	12 (36)
3–4	100 (74)	43 (43)	16 (16)	28 (28)
N/A	3 (2)	1 (33)	0 (0)	1 (33)
*N category*				
0–2	62 (46)	33 (53)	11 (18)	22 (35)
3	51 (34)	28 (55)	10 (20)	19 (37)
*Histology*				
Squamous cell carcinoma	56 (41)	17 (30)	2 (4)	15 (27)
Adenocarcinoma	69 (51)	39 (57)	18 (26)	22 (32)
Not otherwise specified (NOS)	11 (8)	5 (45)	1 (9)	4 (36)
*Immunotherapy (ICI)*				
Yes	36 (26)	16 (44)	5 (14)	11 (31)
No	100 (74)	45 (45)	16 (16)	30 (30)
*Type of ICI*				
Nivolumab	11 (31)	6 (55)	2 (18)	4 (36)
Durvalumab	25 (69)	10 (40)	3 (12)	7 (28)
*PD-L1-status ≥* *1%*				
Yes	39 (29)	16 (41)	5 (13)	11 (28)
No	8 (6)	2 (25)	2 (25)	1 (13)
NA	89 (65)	43 (48)	14 (16)	29 (33)
*Radiation technique*				
VMAT	82 (60)	37 (45)	10 (12)	27 (33)
Step-and-shoot IMRT	19 (14)	9 (47)	4 (21)	5 (26)
3D-conformal RT	35 (26)	15 (43)	7 (20)	9 (26)
*Planning target volume*				
< 700 cc	68 (50)	35 (51)	14 (21)	21 (31)
≥ 700 cc	68 (50)	26 (38)	7 (11)	20 (29)
*Median SUVmax*				
≥ 13.75	59 (43)	24 (41)	9 (15)	16 (27)
< 13.75	59 (43)	27 (46)	11 (19)	16 (27)

Baseline positron emission tomography (PET) and computed tomography (CT) staging were performed in 133 (97.8%) patients before initiation of multimodality treatment to improve contouring quality [13, 14] and cranial contrast-enhanced MRI was acquired in 88 (64.7%) patients, while all other patients obtained cranial contrast-enhanced CT.

cCRT was recommended in a multidisciplinary tumor board for each patient. An Eastern Cooperative Oncology Group (ECOG) performance status (PS) of 0–1 and adequate lung function (diffusing capacity of the lung for carbon monoxide corrected for hemoglobin (DLCO) ≥ 40%, forced expiratory volume in 1 s (FEV1) ≥ 1 L) were required.

Patients were positioned supine with their arms held overhead by WingSTEP™ (Innovative Technologie Voelp, Innsbruck, Austria). Target volumes were defined according to an internal protocol closely following the later published guidelines of the European Society for Radiotherapy and Oncology-Advisory Committee on Radiation Oncology Practice (ESTRO-ACROP) [15] and based conventional planning CT and PET-CT.

Gross tumor volume (GTV) and planning target volume (PTV) were transcribed from the original treatment plans. To differentiate small vs. large volumes, we chose median dichotomization for GTV (78.0 cc) and split PTV in < vs ≥ 700 cc based on literature research of previously published data [16–19].

Prior to the introduction of intensity-modulated radiation therapy (IMRT), TRT with 50 Gy in 2 Gy single-dose fractions, followed by a sequential 16-Gy boost, was used. After implementation of IMRT, TRT consisted of 30 fractions with simultaneous integrated boost (SIB) of 2.0/2.12 Gy to the lymph nodes (LN)/GTV of the primary tumor (PT) with a total dose of 60.0/63.6 Gy.

Follow-up was performed every three months for the first two years after treatment, every six months for the next two years, and annually thereafter. Routine blood tests, pulmonary function tests, clinical examinations, and imaging such as PET-CT or CT scans were analyzed, response was assessed according to RECIST 1.1. Additional diagnostic measures such as contrast-enhanced magnetic resonance imaging (MRI) and bone scintigraphy, were performed when deemed necessary.

Brain metastases (BM), extracranial distant metastases (ecDM) and distant metastases (DM) were documented with PET-CT, CT or MRI scans. Histological confirmation of progressive disease was not obligatory.

The impact of each parameter on BMFS, ecDMFS and DMFS was analyzed by means of Kaplan-Meier analysis using the log-rank test. Multivariable Cox-regression analysis was performed with all parameters which had shown to be significant (*p* < 0.05) in univariate analysis. All analyses including univariate and multivariate analysis were performed using SPSS version 28 (IBM; Armonk, New York, USA).

## Results

A total of 136 consecutive patients with unresectable stage IIIA–C NSCLC (UICC 8th edition) received initial treatment between 2011 and 2020 and were eligible for this analysis. A summary of patient- and tumor-characteristics is shown in Table 1.

In the entire cohort, the median follow-up was 49.7 (range: 0.7–126.1) months. Seventy-eight (57%) patients were older than 65 years and the mean age was 66.9 (range 33.6–82.5) years. Table 2 shows OS, BMFS, ecDMFS and DMFS for the entire cohort and each subgroup.

**Table 2:** Survival parameters

	Median (months)	Median by Subgroups (months)			Number of Patients (%) Entire Cohort		
	Entire cohort	A (2011–2014)	B (2015–2017)	C (2018–2020)	12-months	24-months	36-months
Folllow up	49.7 (44.7–54.7)	117.1 (112.0–122.4)	60.8 (53.7–67.9)	41.7 (38.3–45.1)	–		
BMFS	24.7 (18.3–31.1)	14.9 (9.9–20.0)	20.6 (9.9–31.4)	NR	92 (68)	63 (46)	45 (33)
ecDMFS	23.2 (12.6–33.7)	16.3 (11.1–21.6)	12.9 (6.4–19.5)	NR	85 (63)	60 (44)	42 (31)
DMFS	16.3 (11.4–21.3)	14.7 (9.2–20.3)	12.7 (5.6–19.9)	36.4 (21.8–51.1)	77 (57)	51 (38)	35 (26)
OS	31.2 (16.9–45.6)	19.9 (10.7–29.1)	23.4 (13.0–29.1)	50.5 (36.0–65.1)	101 (74)	72 (53)	52 (38)

**Fig. 1:**
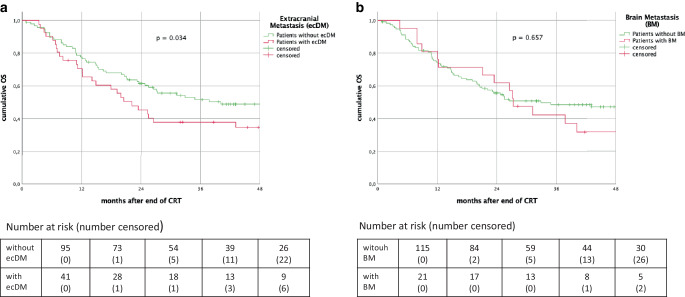
Kaplan–Meier curves of overall survival for all patients with and without extracranial metastasis (ecDM) (**a**) vs. with and without brain metastasis (BM) (**b**)

The median overall survival for patients with DM as FSMP vs. without DM was 25.5 (95% CI: 20.1–31.0) vs. 64.9 (95% CI: 34.3–95.4) months (*p* = 0.037). Median survival after onset of DM as FSMP was 11.9 months (range: 1.0–121). The overall survival of patients with isolated BM as FSMP was 27.4 (95% CI: 20.6–34.1) vs. 32.9 (95% CI: 11.7–54.0) months without BM (*p* = 0.657). Patients survived a median of 13.3 months after the diagnosis of BM (range: 1.3–121). Overall survival in patients presenting with ecDM as FSMP vs. without ecDM was 22.1 (95% CI: 14.4–29.8) vs. 40.1 (95% CI: 18.7–61.3) months (p = 0.034). These patients survived a median of 8.6 months (range: 1–98months). See also Fig. 1.

In univariate analysis for the entire cohort, stratification into treatment groups A, B, and C was significantly associated with BMFS (*p* = 0.004), ecDMFS (*p* = 0.001), and DMFS (*p* = 0.016). Median BMFS was 14.9 (95% CI: 9.9–19.9) months in subgroup A, 20.6 (95% CI: 9.6–31.3) months in subgroup B and was not reached in subgroup C. The median ecDMFS was 16.3 (95% CI: 11.1–21.6), 12.9 (95% CI: 6.4–19.5) months and not reached, for groups A, B and C, respectively. Median DMFS for subgroups A and B was 14.7 (95% CI: 9.2–20.6) and 12.7 (95% CI: 5.6–19.9) months, and for subgroup C was 36.4 (95% CI: 21.8–51.1) months (see also supplementary figures 1–3).

Both, patients treated with simultaneous + maintenance nivolumab and patients treated with maintenance durvalumab were included in this retrospective cohort. We found no significant differences for OS (*p* = 0.841), PFS (*p* = 0.764), DMFS (*p* = 0.919), ecDMFS (*p* = 0.628) and BMFS (*p* = 0.668) between these subgroups. Therefore, we pooled them as an ICI cohort versus patients treated without ICI.

In patients who received ICI, median BMFS was 20.6 (95% CI: 14.4–26.9) months vs. 17.6 (95% CI: 12.9–22.3) for those without ICI (*p* = 0.005). Median ecDMFS was 16.9 (95% CI: 10.2–23.6) months vs. NR, respectively (*p* = 0.003). Median DMFS was 14.9 (95% CI 10.7–19.2) months vs. NR (*p* = 0.031). Median BMFS of patients over 65 was 20.1 (95% CI: 12.6–27.5) months vs. 25.6 (95% CI: 8.7–42.4) months in patients under the age of 65 (*p* = 0.016).

Patients who had a cMRI scan prior to treatment showed no significant difference in DMFS (*p* = 0.295) or BMFS (*p* = 0.189).

We analyzed the use of VMAT and observed significantly longer metastasis free survival in patients that received VMAT vs. patients with 3D-TRT. Median BMFS was 52.2 (95% CI: 22.1–82.3) vs. 14.9 (95% CI: 9.4–20.4) months (*p* = 0.001). Median ecDMFS was not reached vs. 14.9 (95% CI: 7.9–21.9) months (*p* = 0.001) and median DMFS was 25.6 (95% CI: 9.1–42.0) vs. 11.9 (95% CI: 6.7–17.2) months, respectively (*p* = 0.006).

GTV had significant impact on outcome in univariate analysis: DMFS was 26.3 (95% CI: 12.6–40.0) vs 10.8 (95% CI: 8.6–13.0) months (*p* = < 0.001), BMFS was 52.2 (95% CI: 20.2–84.2) vs 14.2 (95% CI: 10.5–17.8) months (*p* < 0.001) and ecDMFS was 64.9 (95% CI: 23.6–106.2) vs 12.7 (95% CI: 8.9–16.6) months (*p* < 0.001) for patients with GTV < 78 cc vs ≥ 78 cc, respectively. In the non-ICI cohort GTV had a significant negative impact on DMFS (*p* = 0.006), BMFS (*p* = 0.004) and ecDMFS (*p* = 0.007). In patients treated with ICI only BMFS (*p* = 0.030) was significantly impacted by GTV whereas DMFS (*p* = 0.074) and ecDMFS (*p* = 0.078) were not.

PTV ≥ 700 cc was significantly associated with shorter BMFS and ecDMFS. The median BMFS for patients with PTV ≥ 700 cc was 16.3 (95% CI: 11.0–21.7) compared to 50.3 (95% CI: 23.5–77.1) months for patients with PTV < 700 cc (*p* = 0.021). The median ecDMFS was 12.7 (95% CI: 8.6–16.8) vs. 55.2 (95% CI: 18.0–92.4) months (*p* = 0.008).

For the entire cohort, patients with a total lung V20 ≥ 30% had a median BMFS of 10.9 months (95% CI: 2.1–19.8) compared to 26.5 months (95% CI: 10.9–42.2) for patients with V20 < 30% (*p* = 0.001). The median ecDMFS was 10.6 months (95% CI: 6.2–14.9) vs. 27.2 months (95% CI: 15.1–39.3) for patients with V20 ≥ 30% and V20 < 30%, respectively (*p* = 0.005), and the median DMFS was 5.6 months (95% CI: 0.0–13.7) vs. 17.9 months (95% CI: 11.6–24.3) (*p* = 0.004).

In patients treated without ICI, stratification by year of treatment showed significant association with ecDMFS (*p* = 0.004) and DMFS (*p* = 0.047) and a trend toward longer BMFS (*p* = 0.061). In the non-ICI cohort, a significant association was found between the use of VMAT and BMFS (*p* = 0.021) and ecDMFS (0.013). For DMFS there was a trend (*p* = 0.054). The median BMFS was 14.1 months (95% CI: 10.1–18.3) for patients with PTV ≥ 700 cc and 24.7 months (95% CI: 12.1–37.4) for patients with PTV < 700 cc (*p* = 0.036). The median ecDMFS was 11.9 months (95% CI: 10.2–13.6) and 27.4 months (95% CI: 20.8–33.9), respectively (*p* = 0.006).

The median BMFS amounted to 11.2 months (95% CI: 4.6–17.8) for patients with SUVmax ≥ 13.75 vs. 21.1 months (95% CI: 13.9–28.3), for patients with SUVmax < 13.75 (*p* = 0.006). The median ecDMFS was found to be significantly longer in patients with a lower V20 (< 30) than those with a higher V20 (≥ 30). The same was observed for median DMFS and BMFS. Patients with higher SUVmax (≥ 13.75) also showed a trend towards shorter median DMFS. The *p*-values for these associations were 0.009 and 0.063, respectively.

V20 ≥ 30 was significantly associated with DMFS, ecDMFS and BMFS in the non-ICI cohort: Median BMFS was not reached for V20 ≥ 30 (*p* = 0.002) and V20 < 30. In Non-ICI patients, the median ecDMFS (*p* = 0.009) and the median DMFS (*p* = 0.020) were also significantly linked to V20 ≥ 30. The detailed results of the univariate analyses can be found in Table 3.

**Table 3:** Univariate analysis

Patient cohort	Parameter	Univariate analysis (*p*-value)		
		BMFS	ecDMFS	DMFS
Entire cohort (*n* = 136)	ICI	0.005	0.003	0.031
	PD-L1 pos	0.006	0.002	0.036
	Treatment group	0.004	0.001	0.016
	Age ≥ 65	0.016	0.174	0.245
	Histology	0.894	0.507	0.602
	T status	0.218	0.258	0.024
	N status	0.940	0.749	0.412
	V20 ≥ 30	< 0.001	0.005	0.004
	GTV ≥ 78 cc	< 0.001	< 0.001	< 0.001
	PTV ≥ 700 cc	0.021	0.004	0.093
	SUVmax ≥ 13.75	0.369	0.056	0.290
	cMRI	0.189	–	0.295
	VMAT	0.001	0.001	0.006
Non-ICI (*n* = 100)	PD-L1 pos	0.055	0.030	0.033
Non-ICI (*n* = 100)	Treatment group	0.061	0.004	0.047
	Age ≥ 65	0.119	0.434	0.637
	Histology	0.976	0.647	0.253
	T status	0.052	0.209	0.010
	N status	0.894	0.733	0.597
	V20 ≥ 30	0.002	0.009	0.020
	GTV ≥ 78 cc	0.004	0.007	0.006
	PTV ≥ 700 cc	0.036	0.022	0.144
	SUVmax ≥ 13.75	0.158	0.006	0.209
	VMAT	0.021	0.013	0.054
*ICI*				
(*n* = 36)	PD-L1 pos	0.833	0.759	0.631
	Type of ICI	0.668	0.628	0.919
	Treatment group	0.503	0.415	0.746
	Age ≥ 65	0.172	0.600	0.388
	Histology	0.809	0.588	0.480
	T status	0.834	0.417	0.239
	N status	0.799	0.651	0.564
	V20 ≥ 30	0.971	0.944	0.314
	GTV ≥ 78 cc	0.030	0.078	0.074
	PTV ≥ 700 cc	0.516	0.343	0.509
	SUVmax ≥ 13.75	0.670	0.714	0.526
	VMAT	All patients	Treated	With VMAT

The multivariate analysis for the entire cohort included use of immune checkpoint inhibitors (ICI), PD-L1-status ≥ 1%, stratification by treatment year, age over 65, T‑stage, V20 ≥ 30, GTV ≥ 78 cc PTV ≥ 700 cc and use of VMAT.

V20 ≥ 30 was significantly associated with BMFS [HR: 2.400 (95% CI: 1.396–4.126, *p* = 0.002)], and DMFS [HR: 1.784 (95% CI: 1.028–3.097, *p* = 0.040)].

GTV ≥ 78 cc had a significant negative impact on BMFS [HR: 2.100 (95% CI: 1.396–3.358, *p* = 0.002)], ecDMFS [HR: 1.739 (95% CI: 1.006–2.835, *p* = 0.027)] and DMFS [HR: 2.394 (95% CI: 1.485–3.858, *p* < 0.001)].

BMFS was significantly associated with Age ≥ 65 [HR: 1.629 (95% CI: 1.031–2.572, *p* = 0.036)].

In patients treated without ICI, a GTV ≥ 78 cc was also associated with shorter BMFS [HR: 1.976 (95% CI: 1.181–3.308, *p* = 0.010)] and DMFS [HR: 2.049 (95% CI: 1.181–3.554, *p* = 0.011)].

SUVmax ≥ 13.75 remained a predictor of short ecDMFS [HR: 2.420 (95% CI: 1.379–4.248, *p* = 0.002)] V20 ≥ 30 was significantly associated with shorter ecDMFS [HR: 2.883 (95% CI: 1.512–5.497, *p* = 0.001)] and BMFS [HR: 2.273 (95% CI: 1.301–3.970, *p* = 0.004)].

For detailed results of the multivariate analysis refer to Table 4.

**Table 4:** Multivarate analysis

Patient	Parameter	Multivariate analysis					
		BMFS (HR (95% CI)	*p*	ecDMFS HR (95% CI)	p	DMFS (HR (95% CI)	p
Entire	ICI	0.720 (0.356–1.455)	0.360	0.609 (0.279–1.330)	0.214	0.802 (0.429–1.498)	0.489
Cohort	PD-L1 pos	1.194 (0.679–2.099)	0.538	1.427 (0.783–2.599)	0.246	1.176 (0.726–1.905)	0.509
*N* = 136	Treatment groups	1.150 (0.688–1.923)	0.594	0.828 (0.482–1.422)	0.495	1.122 (0.692–1.820)	0.641
	Age ≥ 65	*1.629 (1.031–2.572)*	*0.036*	–	–	–	–
	T Stage	**–**	**–**	–	–	*0.789 (0.632–0.985)*	*0.036*
	V20 ≥ 30	*2.400 (1.396–4.126)*	*0.002*	1.738 (0.992–3.046)	0.053	*1.784 (1.028–3.097)*	*0.040*
	GTV ≥ 78 cc	*2.100 (1.396–3.358)*	*0.002*	*1.739 (1.006–2.835)*	*0.027*	*2.394 (1.485–3.858)*	*<* *0.001*
	PTV ≥ 700 cc	1.160 (0.729–1.846)	0.531	1.427 (0.879–2.316)	0.150	**–**	**–**
	VMAT	0.631 (0.296–1.346)	0.234	0.947 (0.422–2.126)	0.895	0.665 (0.321–1.375)	0.271
Non-ICI	PD-L1 pos	–	–	1.058 (0.492–2.278)	0.885	1.442 (0.817–2.545)	0.207
*n* = 100	Treatment groups	–	–	0.882 (0.482–1.615)	0.684	0.956 (0.661–1.382)	0.811
	SUVmax > 13.75	–	–	*2.420 (1.379–4.248)*	*0.002*	–	–
	T Stage	**–**	**–**	**–**	**–**	0.831 (0.651–1.060)	0.136
	V20 ≥ 30	*2.273 (1.301–3.970)*	*0.004*	*2.883 (1.512–5.497)*	*0.001*	1.688 (0.952–2.994)	0.073
	GTV ≥ 78 cc	*1.976 (1.181–3.308)*	*0.010*	1.611 (0.907–2.689)	0.104	*2.049 (1.181–3.554)*	*0.011*
	PTV ≥ 700 cc	1.157 (0.693–1.931)	0.576	1.499 (0.836–2.689)	0.174	1.030 (0.638–1.661)	0.905
	VMAT	0.715 (0.428–1.194)	0.200	0.821 (0.308–2.184)	0.692	–	–

The median post-progression survival in the entire cohort after onset of DM was 13.3 (95% CI: 6.0–20.6) months. Patients initially treated with ICI sowed a clear trend towards longer post-DM-survival of 20.7 (95% CI: 13.9–27.6) vs 7.8 (95% CI: 4.4–11.5) months (*p* = 0.052). The median survival after onset of DM in subgroup A, B and C were 7.2 (95% CI: 3.1–11.3), 7.8 (95% CI: 0.0–24.2) and 18.5 (95% CI 12.1–25.0) months, respectively (*p* = 0.243).

After BM patients in the entire cohort survived a median of 13.3 (95% CI: 6.4–20.2) months. Median post-BM-survival was not reached in patients who received ICI in the course of their initial treatment compared to 9.5 (95% CI: 2.0–16.9) months in those without ICI (*p* = 0.177). For subgroups A, B and C median post-BM-survival was 9.5 (95% CI: 0.0–22.6), 7.8 (0.0–17.9) and 13.3 (95% CI: 10.7–6.0) months, respectively (*p* = 0.787).

The median post-ecDM-survival was 8.6 (95% CI: 1.6–15.5) months in the entire cohort and 20.7 (96% CI: 15.4–26.1) vs 6.4 (95% CI: 3.5–9.4) months for patients initially treated with vs without ICI, respectively. In subgroup A it was 4.8 (95% CI: 1.1–8.5) months, in subgroup B it was 6.8 (95% CI: 0.0–27.7) months and in subgroup C it was 18.6 (95% CI: 2.1–35.1) months (*p* = 0.357).

## Discussion

In the past decade, important advances have been achieved in the multimodal treatment of inoperable stage III NSCLC. The introduction of VMAT has decreased the toxicity of TRT [20–22] and routine concurrent chemo-radiotherapy has constantly been improved [23]. The PACIFIC trial has changed the landscape of thoracic oncology with unprecedented improvements in OS and PFS [7, 8, 24]. In this comprehensive analysis of patients treated in the past decade we depict patterns of first distant failure after cCRT for Stage III NSCLC. We identified factors during initial treatment that predict patients’ risk for later onset of metastasis and analyzed impact of metastasis on OS of affected patients.

We observed no significant differences for OS, PFS, DMFS, ecDMFS and BMFS between patients treated with durvalumab vs. those treated with nivolumab, therefore we decided pool both cohorts together against patients treated without ICI to raise patient numbers.

In our cohort the median DMFS for patients treated with CRT+ICI was 29.5 months vs 14.93 months (*p* = 0.031) for those treated with CRT alone. This is in close accordance with the data of the PACIFIC-trial, were the median time to death or distant metastasis was reported as 28.3 months for patients treated with durvalumab vs.16.2 months for patients in the placebo arm [25]. In the LUN 14–179 trial, a time to metastatic disease or death of 30.7 months (95% CI: 18.7 to NR) was reported for patients receiving pembrolizumab after cCRT [26]. In contrast to Kishi et al., we did not observe female sex as a negative prognosticator for the onset of DM [27].

As previously described, the implementation of Durvalumab maintenance drastically improves intrathoracic control after cCRT [7, 8, 24, 28–33]. In univariate analysis for BMFS, ecDMFS and DMFS we observed vast improvements in patients treated with cCRT+ICI vs those treated with cCRT alone (*p* = 0.005; *p* = 0.003 and *p* = 0.031) as shown in Table 3 and Fig. 1. Kishi et al. recently reported significantly lower rates of distant metastasis in patients treated with cCRT + durvalumab vs. without durvalumab in a real-world setting [30].

BMFS and ecDMFS had a significant negative correlation with larger GTV, PTV (≥ 700 cc) and with total lung V20 ≥ 30%. This suggests that patients with initially larger initial tumor burden are more likely to experience distant failure after cCRT. The association of larger PTVs with diminished regional control has been reported frequently reported in literature [18, 19, 34, 35]. However, the impact of initial GTV and its’ shrinkage during CRT in patients that afterwards receive IO maintenance is currently unknown and should be considered for future investigations [36, 37]. Also, underdosage of large tumours or reduced margins at the discretion of the performing physicians could be an explanation of this strong negative correlation.

The study split patients into three subgroups (A 20112014, B 2015–2017 and C 2018–2020) and showed constant improvements of DMFS, BMFS and ecDMFS over the years, especially in subgroup C, which coincided with the implementation of ICItreatment Interestingly, the study also observed numerical improvements in post-DM-survival from 7.2 months in subgroup A to 15.1 months in subgroup B to 18.5 in subgroup C. Although these numerical benefits were not statistically significant, they hint towards improved post-progression therapy and we are planning further analyses on this subject [4, 38].

Patients treated with cCRT alone, who initially presented with PD-L1 expressing (≥ 1%) tumors had significantly worse DMFS in univariate analysis (*p* = 0.033) and showed a clear negative trend in multivariate analysis (HR: 1.666; *p* = 0.091). This indicates a vast improvement in outcomes for those patients after implementation of durvalumab maintenance, considering IO was almost exclusively administered in patients with PD-L1 on ≥ 1% of tumor cells due to regulations by the European medicines agency (EMA).

The importance of post progression survival (PPS) has been highlighted by Imai et al. [39]. In this study’s patients initially treated with cCRT+ICI showed numerically improved post-DM-survival compared to those who were initially treated with cCRT alone. Interestingly, BM as FSMP did not significantly impact survival, whereas ecDM lead to impaired OS. Also PPS after BM as FSMP was significantly longer than after the onset of ecDM. This could be due to the highly efficient treatment options of BM via stereotactic radiosurgery (SRS) [40]. Overall the median post progression survival achieved in our cohort (13.3 months) is 2.7 months longer compared to the 10.6 months reported by Delasos et al., but nevertheless unsatisfying [41]. It is of utmost interest to further improve treatment for patients with progression after cCRT (+/−) ICI for stage III NSCLC, especially those with ecDM as FSMP. Further trials are needed to tailor post progression therapy aiming to improve PPS.

We are planning to analyse factors for PPS, like second line TKI-therapy, ICI-therapy or chemotherapy in the near future.

Given the limitations of this retrospective study we want to acknowledge the low number of patients with PD-L1 assessment prior to treatment, lack of Next Generation Sequencing and subtotal MRI staging prior to treatment. These points should be included in all prospective trials concerning lung cancer.

## Conclusion

In conclusion, this retrospective analysis of unresectable stage III NSCLC patients identified V20 ≥ 30%, GTV ≥ 78 cc, and age over 65 as strong prognosticators for BMFS and GTV ≥ 78 cc for ecDMFS. Additionally, T‑stage, V20 ≥ 30 and GTV ≥ were found to be prognosticators for DMFS. Implementation of ICI treatment resulted in a significant improvement of BMFS, ecDMFS, and DMFS. Patients with ecDM had shorter overall survival, while BM did not impact survival of affected patients compared to the entire cohort.

### Supplementary Information

sFigure 1: Kaplan–Meier curves of brain metastasis-free survival (BMFS), for all patients (left) vs. patients without ICI (right) stratified by treatment year groups

sFigure 2: Kaplan–Meier curves of extracranial distant metastasis-free survival (ecDMFS) for all patients (left) vs. patients without ICI (right) stratified by treatment year groups

sFigure 3: Kaplan–Meier curves of distant metastasis-free survival (DMFS) for all patients (left) vs. patients without ICI (right) stratified by treatment year groups

sFigure 4: Kaplan–Meier curves of extracranial distant metastasis-free survival (ecDMFS) for all patients with and without ICI

sFigure 5: Kaplan–Meier curves of brain metastasis-free survival (BMFS), for all patients with and without ICI
