# Supplementary figures and images for: First-site-metastasis pattern in patients with inoperable stage III NSCLC treated with concurrent chemoradiotherapy with or without immune check-point inhibition: a retrospective analysis
Source: Strahlenther Onkol. 2023 Nov 17;200(7):614–23. doi: 10.1007/s00066-023-02175-6 (PMC11186867; doi:10.1007/s00066-023-02175-6)

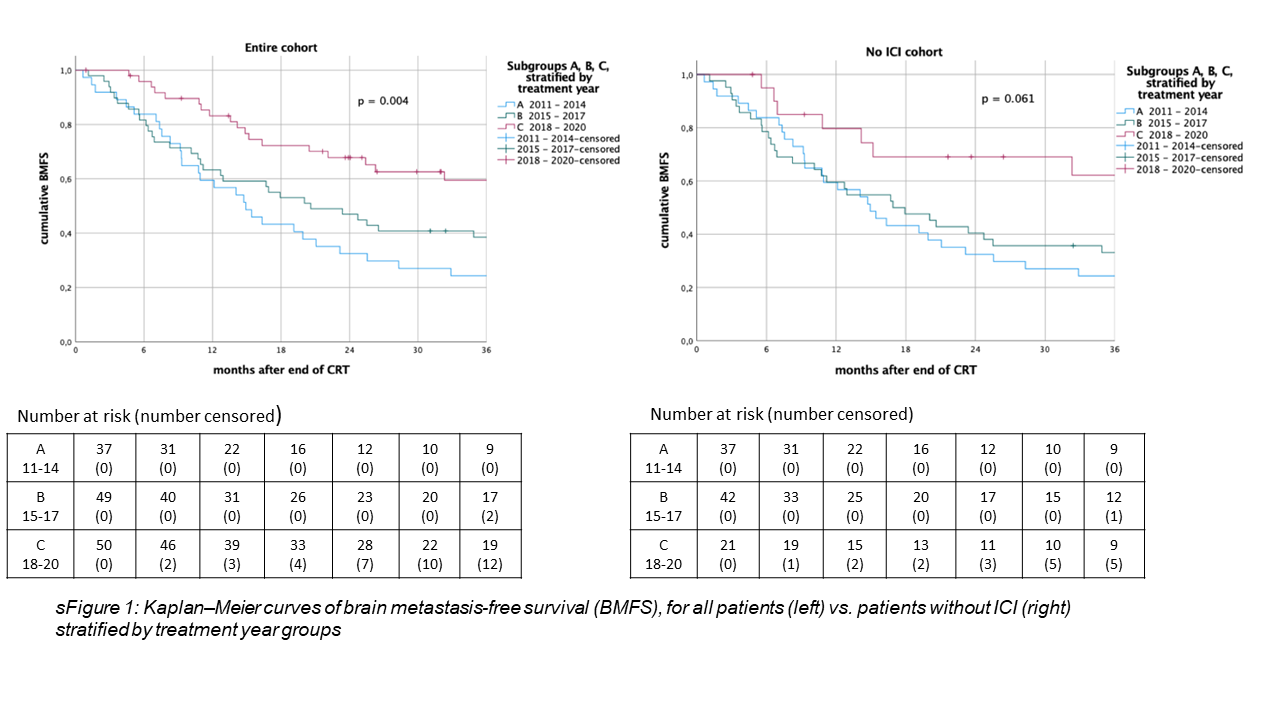

Supplement: Supplementary file 1 — sFigure 1: Kaplan–Meier curves of brain metastasis-free survival (BMFS), for all patients (left) vs. patients without ICI (right) stratified by treatment year groups [file 66_2023_2175_MOESM1_ESM.tif]

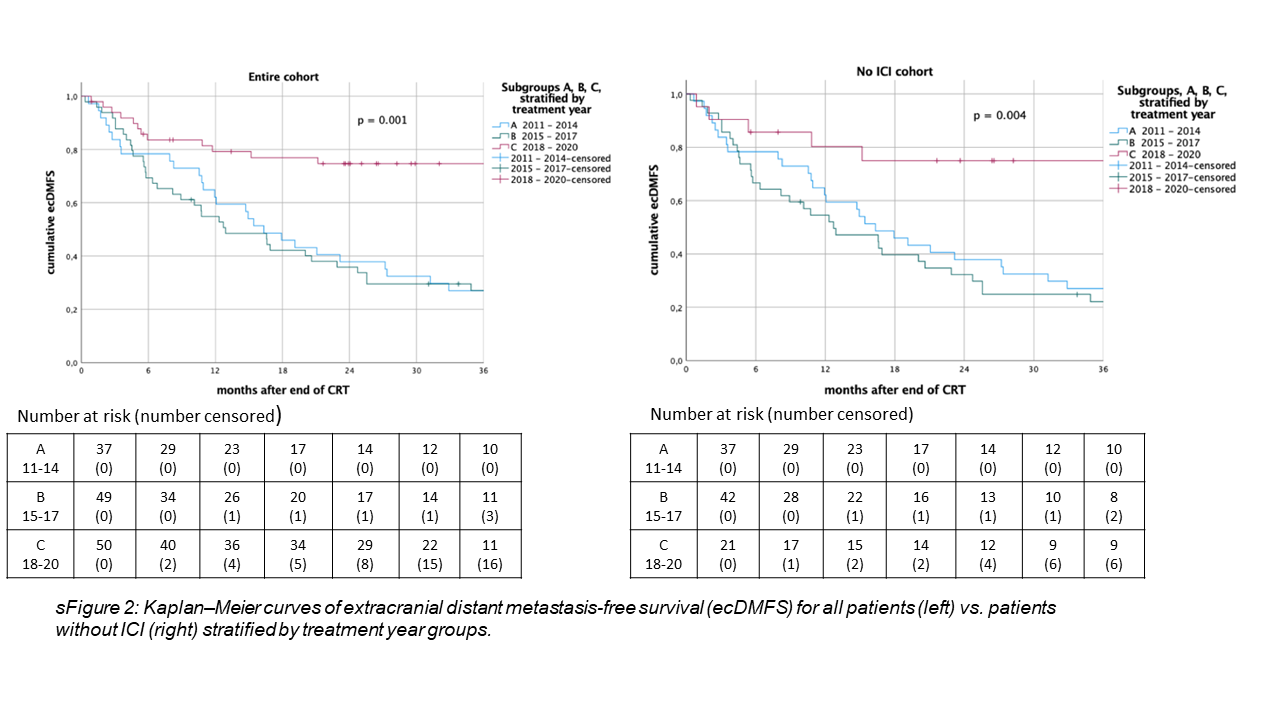

Supplement: Supplementary file 2 — sFigure 2: Kaplan–Meier curves of extracranial distant metastasis-free survival (ecDMFS) for all patients (left) vs. patients without ICI (right) stratified by treatment year groups [file 66_2023_2175_MOESM2_ESM.tif]

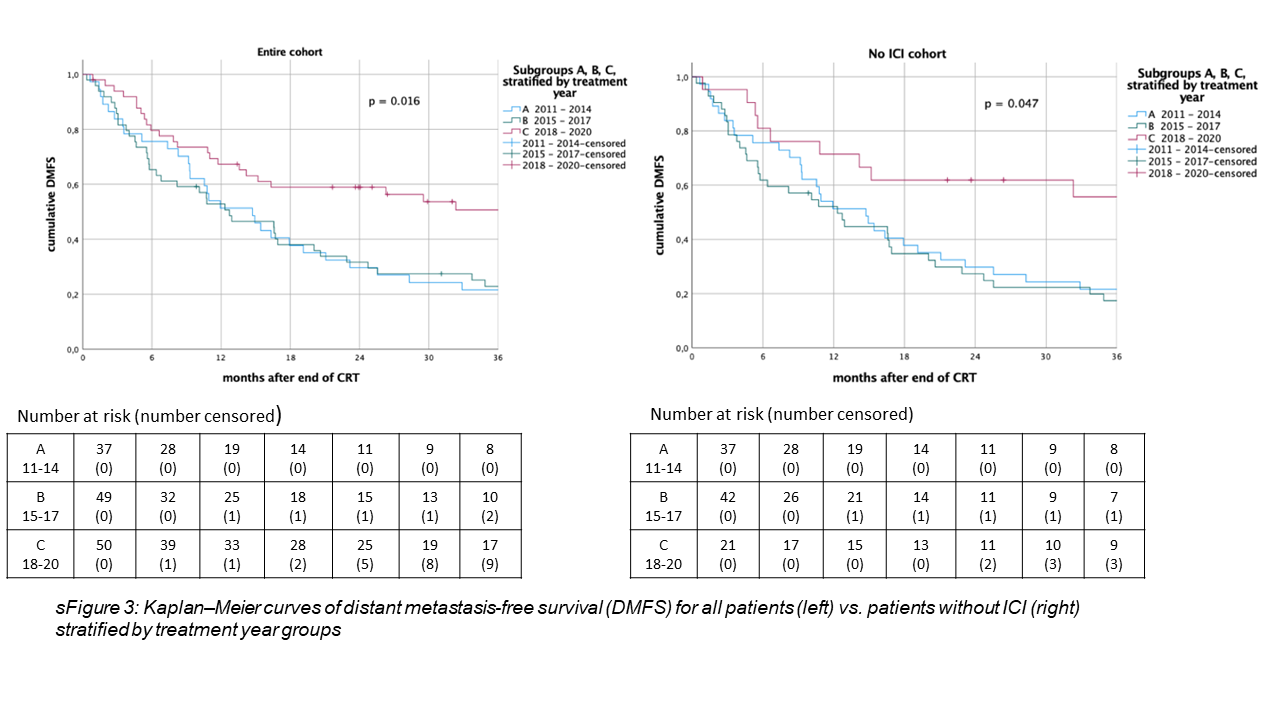

Supplement: Supplementary file 3 — sFigure 3: Kaplan–Meier curves of distant metastasis-free survival (DMFS) for all patients (left) vs. patients without ICI (right) stratified by treatment year groups [file 66_2023_2175_MOESM3_ESM.tif]

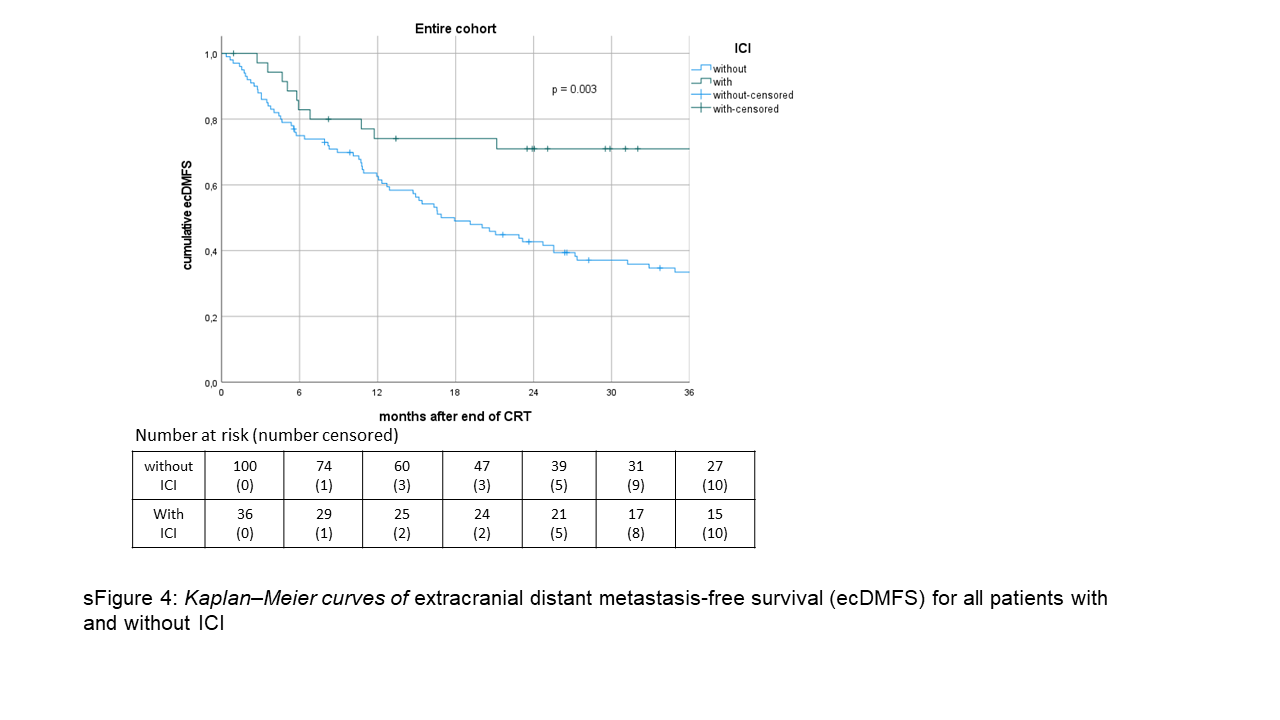

Supplement: Supplementary file 4 — sFigure 4: Kaplan–Meier curves of extracranial distant metastasis-free survival (ecDMFS) for all patients with and without ICI [file 66_2023_2175_MOESM4_ESM.tif]

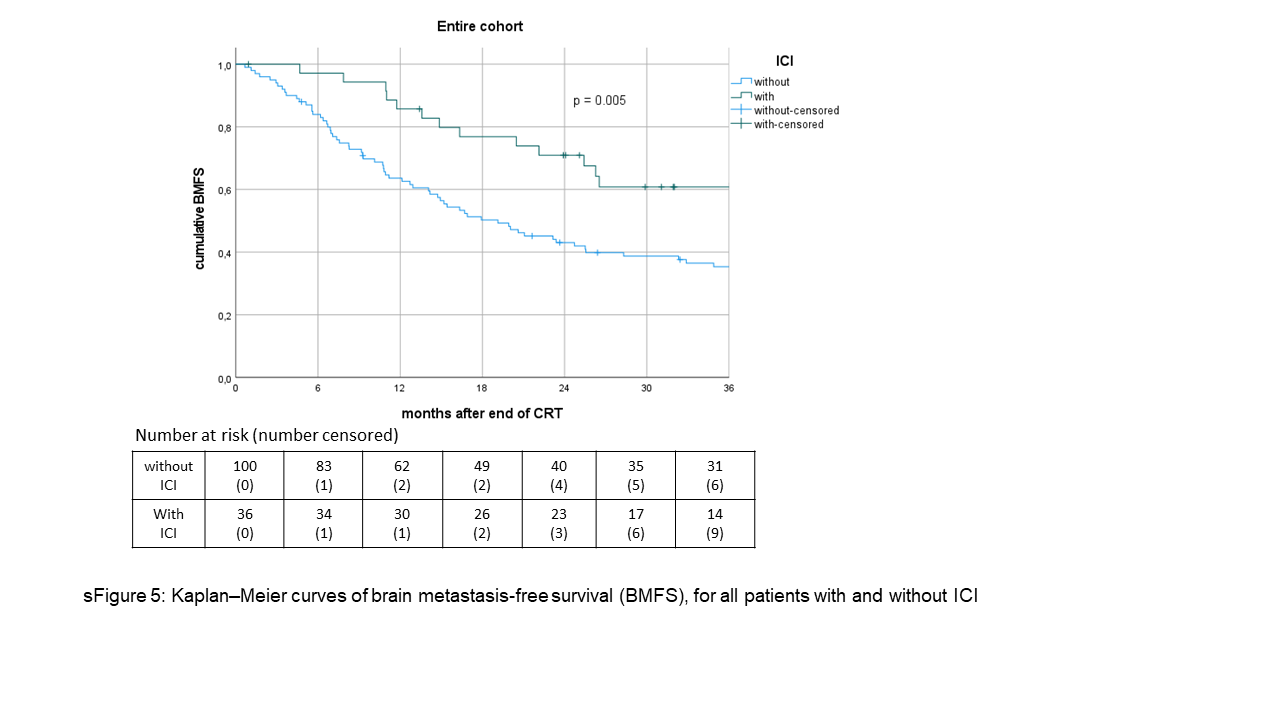

Supplement: Supplementary file 5 — sFigure 5: Kaplan–Meier curves of brain metastasis-free survival (BMFS), for all patients with and without ICI [file 66_2023_2175_MOESM5_ESM.tif]
